# Assessment of Health Conditions and Health Service Use Among Transgender Patients in Canada

**DOI:** 10.1001/jamanetworkopen.2020.15036

**Published:** 2020-08-28

**Authors:** Alex Abramovich, Claire de Oliveira, Tara Kiran, Tomisin Iwajomo, Lori E. Ross, Paul Kurdyak

**Affiliations:** 1 Institute for Mental Health Policy Research, Centre for Addiction and Mental Health, Toronto, Ontario, Canada; 2 Dalla Lana School of Public Health, University of Toronto, Toronto, Ontario, Canada; 3 ICES, Toronto, Ontario, Canada; 4 Centre for Health Economics and Hull York Medical School, University of York, Heslington, York, United Kingdom; 5 Institute of Health Policy, Management, and Evaluation, University of Toronto, Toronto, Ontario, Canada; 6 MAP Centre for Urban Health Solutions, Department of Family and Community Medicine, St Michael’s Hospital, Toronto, Ontario, Canada; 7 Department of Family and Community Medicine, Faculty of Medicine, University of Toronto, Toronto, Ontario, Canada; 8 Quality Business Unit, Ontario Health, Toronto, Ontario, Canada; 9 Department of Psychiatry, Faculty of Medicine, University of Toronto, Toronto, Ontario, Canada

## Abstract

**Importance:**

Transgender individuals experience stigma, discrimination, and socioeconomic disadvantages, leading to a myriad of poor health outcomes and high rates of disease burden; however, transgender health continues to be an understudied area.

**Objective:**

To examine sociodemographic characteristics, health conditions, and health service utilization patterns among transgender individuals compared with the general population.

**Design, Setting, and Participants:**

This cross-sectional study of 2085 transgender individuals from 3 large cities in Ontario, Canada, compared characteristics and health service use among transgender individuals with the general population in the province. Transgender individuals were identified through data obtained from 4 outpatient community and hospital clinics, which were linked with health administrative data between January 2012 and December 2016. Data were analyzed between October 2018 and May 2020. Individuals were age-matched 1:5 to a random 5% sample of the general Ontario population (10 425 individuals).

**Main Outcomes and Measures:**

Sociodemographic variables, health service use, and chronic conditions among transgender individuals and the general population were compared.

**Results:**

This study included a sample of 2085 transgender individuals with a mean (SD) age of 30.40 (12.81) years; 771 (37.0%) identified as transgender women. Compared with 10 425 cisgender controls, trangender individuals were more likely to live in lower-income neighborhoods (lowest-income quintile: 625 [30.0%] vs 2197 [21.1%]; *P* < .001) and experience chronic physical and mental health conditions, including higher rates of asthma (489 [23.5%] vs 2034 [19.5%]; *P* < .001), diabetes (115 [5.5%] vs 352 [3.4%]; *P* < .001), chronic obstructive pulmonary disease (51 [2.4%] vs 156 [1.5%]; *P* < .001), and HIV (34 [1.6%] vs 12 [0.1%]; *P* < .001). Comorbid chronic health conditions were higher among the transgender population compared with the cisgender population (702 [33.7%] vs 2941 [28.2%]; *P* < .001). Transgender individuals also had higher health service use compared with the general population, particularly for mental health and self-harm, including mean (SD) number of psychiatrist visits between January 2012 and December 2016 (8.25 [23.13] vs 0.93 [9.57]; standardized difference, 5.84).

**Conclusions and Relevance:**

This study found higher rates of mental and physical comorbidities and higher health service use among transgender individuals compared with cisgender individuals. Further research should explore reasons for these findings. Clinicians caring for transgender individuals should be aware of the high potential for mental health issues and self-harm.

## Key Points

**Question:**

How do sociodemographic characteristics, chronic conditions, and health service utilization trajectories differ among transgender individuals compared with the general population?

**Findings:**

This cross-sectional study identified and linked health administrative data for 2085 transgender individuals in Ontario, Canada, and found that transgender individuals are more likely to live in lower-income neighborhoods, experience chronic health conditions, and have higher health service use than the general population.

**Meaning:**

This study demonstrates the need to improve the capacity to identify transgender individuals in administrative health data to understand the factors underlying their high rates of disease burden.

## Teaser

This cross-sectional study draws from identified and linked and health administrative data in Ontario, Canada, to compare the sociodemographic characteristics and health care use of transgender individuals with the general population of the province.

## Introduction

*Transgender* is an umbrella term that refers to individuals who do not identify with the sex assigned to them at birth (eg, a person who was assigned male at birth but identifies as a woman is a transgender woman). *Gender expansive* refers to a wide range of gender identities and expressions that expand and broaden the definitions of gender-normative identities and are neither male nor female, whereas *cisgender* refers to individuals who identify as the sex assigned to them at birth (eg, a person who was assigned female at birth and identifies as a woman is a cisgender woman). Population studies yielding prevalence data for transgender individuals have estimated that 0.5% to 1.3% of adults are transgender and that there are approximately 25 million transgender individuals worldwide.^1^ Research has consistently shown that transgender individuals are medically underserved and experience ongoing stigma, discrimination, and socioeconomic disadvantages, leading to a myriad of poor health outcomes and high rates of disease burden.^1,2,3,4,5^ However, general health continues to be among the most understudied areas in transgender research.^4^ We must be able to identify and study transgender individuals at a population level to conduct rigorous and meaningful analysis of the transgender population.

Hospital and primary health care settings routinely collect administrative data on sex designation, rather than gender identity, based on what is listed on a person’s provincial health insurance card.^6^ The expectation that every individual will identify with the gender binary creates challenges for transgender and gender-expansive individuals to navigate the health care system and perpetuates institutional erasure. The lack of data collection on gender identity has resulted in limited information on the health conditions that affect the transgender population and how they are being served by the health care system. Data are primarily available through self-reported surveys,^2,6,7,8,9,10,11,12,13^ making it difficult to understand the health care utilization patterns and health outcomes among transgender individuals.

We sought to identify a sample of transgender individuals in administrative data and use the sample to characterize the sociodemographic characteristics, health conditions, and health service use of transgender individuals compared with the general population in 1 Canadian province. We were specifically interested in the prevalence of asthma, cancer, and HIV, based on findings from previous research^4^ that has reported a higher prevalence of these conditions among transgender individuals. In addition, we were interested in mental health service use, given that previous research has reported mental health issues among transgender individuals, resulting from stigma, discrimination, and violence.^1,4,5^ We hypothesized that transgender individuals would have higher rates of mental illness and more emergency department (ED) visits and hospitalizations owing to mental health and self-harm related reasons.

## Methods

### Setting and Context

Ontario is Canada’s most populated province, with approximately 14.5 million people counted in 2019. The Ontario Health Insurance Plan provides health insurance coverage for hospital and physician visits and medically necessary laboratory tests to all permanent residents. Access to transgender-inclusive health care and transgender rights differ across Canada. The Ontario Human Rights Code protects people from discrimination and harassment due to gender identity and gender expression; however, transgender Ontarians report experiences of widespread discrimination in health care settings.^14^

### Study Design

We conducted a cross-sectional study comparing characteristics and service use of transgender individuals with the general population in Ontario, Canada, between January 2012 and December 2016. All sociodemographic variables were calculated as of January 1, 2016; data were analyzed between October 2018 and May 2020. We identified transgender individuals in community health centers and an outpatient hospital clinic and linked their data with population-wide health administrative data held at ICES. ICES is a prescribed entity under section 45 of Ontario’s Personal Health Information Protection Act. Section 45 authorizes ICES to collect personal health information, without consent, for the purpose of analysis or compiling statistical information, including the management, evaluation, monitoring, and allocation of resources, for all or part of the health system. Projects conducted under section 45 by definition do not require review by a research ethics board. This project was conducted under section 45 and approved by ICES’s privacy and legal office. This study followed the Strengthening the Reporting of Observational Studies in Epidemiology (STROBE) reporting guideline.

### Data Sources

The ICES data repository includes individual-level, linkable, longitudinal data on most publicly funded health care services for individuals covered by Ontario health insurance. These data sets were linked using unique encoded identifiers and analyzed at ICES. The Ontario Health Insurance Plan claims database includes data on outpatient care, namely claims for physician services. The Registered Persons Database was used to capture sociodemographic variables and all-cause mortality (if death occurred during the study period). The Immigration, Refugees, and Citizenship Canada Permanent Resident Database was used to determine migrant status. The Ontario Drug Benefit Database was used to determine whether individuals received benefits based on financial need. The National Ambulatory Care Reporting System was used to identify all ambulatory care, including ED visits. The Ontario Mental Health Reporting System was used to identify hospitalizations occurring in mental health–designated hospital beds. The Discharge Abstract Database was used to identify non–mental health hospital admissions. Medical comorbidities were determined by validated chronic condition algorithms based on physician billings and hospital discharge databases available at ICES.^15,16,17,18,19,20^ We identified other psychiatric and medical comorbidity using the Johns Hopkins Adjusted Clinical Group software, version 10.0, which categorizes codes from the ninth and tenth revisions of the *International Classification of Diseases* (*ICD-9*/*ICD-10*) and into diagnosis clusters referred to as Aggregated Diagnosis Groups (ADGs), based on 5 clinical dimensions^21^ (described in eAppendix 1 in the Supplement).

### Identifying Transgender Individuals

Transgender individuals were identified through data obtained from 4 outpatient health clinics across Ontario. Clinics were located in 3 cities (Ottawa, Thunder Bay, and Toronto) and have expertise in working with the transgender population. All 4 clinics regularly collect data on self-defined gender identity, making it possible for them to identify their transgender patients within their clinic health records. Data from each clinic were extracted through an electronic medical record search or manual record audit to identify all transgender patients seen at each clinic between January 2012 and December 2016. The following information was extracted from the clinic medical records for transgender individuals: Ontario health insurance numbers, date of birth, sex listed on health card, and self-defined gender identity. Data were linked to administrative databases held at ICES using Ontario health insurance numbers and date of birth. One of the participating clinics was unable to provide the gender identity information of transgender individuals. Cases in which gender identity was missing were classified as unknown. Because of the small sample sizes in numerous gender identity categories and to protect confidentiality and privacy, self-reported gender identities were collapsed into the following 4 categories: transgender woman or transfeminine, transgender man or transmasculine, nonbinary, and unknown. The full spectrum of gender identities represented in our data can be found in eAppendix 2 in the Supplement.

### Matched Controls

Transgender individuals were matched 1:5 on age to a random 5% sample of the general Ontario population (excluding individuals included in the transgender sample). The comparison sample was meant to only include cisgender individuals; however, there is a possibility that transgender individuals who were not captured in the transgender sample may have been included, given that approximately 0.5% to 1.3% of adults are transgender^1^ and we were not able to identify all transgender individuals in Ontario. The transgender and general population samples included Ontario residents eligible for Ontario health insurance from 2012 to 2016.

### Variables

We measured the following sociodemographic variables for both groups: age, sex listed on health card, neighborhood-level income (measured in quintiles at the census tract level), rurality (derived through postal code), and migrant status (ie, Canadian born, immigrant, refugee). We assessed whether individuals received prescriptions via the public drug plan (Ontario Drug Benefit), a marker of low income. We measured the following medical comorbidities: rheumatoid arthritis, asthma, cancer, chronic health failure, Crohn disease and colitis, chronic obstructive pulmonary disease (COPD), diabetes, HIV, hypertension, and myocardial infarction. Health service variables measured included physician visits (defined as primary care physician visits and measured by total visits); mental health and non-mental health visits and psychiatrist visits; and visits to other specialists such as urologists, plastic surgeons, obstetrician-gynecologists, cardiologists, and endocrinologists. We also measured ED visits by total number of visits, mental health–related ED visits, self-harm–related ED visits, and non–mental health–related ED visits. We examined hospitalizations by total number of hospitalizations, mental health–related hospitalizations, and non–mental health–related hospitalizations.

### Statistical Analysis

Descriptive and baseline characteristics (frequencies and means) were calculated across both samples. Differences between both samples and between gender identity categories were explored using χ^2^ tests, 1-way analysis of variance, and standardized mean differences (SMDs). *P* values and standardized differences were used to test whether groups were statistically different from one another, with significance set to *P* < .05 in 2-tailed tests. All analyses used SAS version 9.4 (SAS Institute).

## Results

The sociodemographic and clinical characteristics of 2085 transgender individuals included in the study compared with a 5% random sample of 10 425 age-matched cisgender individuals are presented in Table 1. Transgender individuals had a mean (SD) age of 30 (12.87) years; most were under the age of 45, with 882 (42.3%) aged 25 to 44 years and 894 (42.9%) younger than 24 years (Table 1). Transgender individuals in the unknown gender identity category had the highest mean (SD) age (32.06 [14.17] years), followed by transgender women (31.36 [13.08] years), transgender men (28.95 [11.94] years), and nonbinary individuals (28.75 [11.29] years) (Table 2).

**Table 1.** Sociodemographic and Clinical Characteristics of Transgender Individuals Compared With Age-Matched Cisgender Individuals

Characteristics	No. (%)			*P* value	SMD	Variance ratio
	Transgender individuals (n = 2085)	Cisgender individuals (n = 10 425)	Total (N = 12 510)			
**Demographic characteristics**						
Age, y						
Mean (SD)	30.25 (12.87)	30.25 (12.86)	30.25 (12.86)	NA	NA	NA
Median (IQR)	26 (21-37)	26 (21-37)	26 (21-37)			
Age group, y						
≤24	894 (42.9)	4470 (42.9)	5364 (42.9)	NA	NA	NA
25-44	882 (42.3)	4410 (42.3)	5292 (42.3)			
45-64	276 (13.2)	1380 (13.2)	1656 (13.2)			
≥65	33 (1.6)	165 (1.6)	198 (1.6)			
Sex listed on health card						
Female	1055 (50.6)	5197 (49.9)	6252 (50.0)	.53	0.01	0.20
Male	1030 (49.4)	5228 (50.1)	6258 (50.0)		0.01	0.20
Nearest census-based neighborhood income^a^						
1 (low)	625 (30.0)	2197 (21.1)	2822 (22.6)	<.001	0.21	0.16
2 (medium-low)	417 (20.0)	2036 (19.5)	2453 (19.6)		0.01	0.20
3 (medium)	368 (17.6)	2093 (20.1)	2461 (19.7)		0.06	0.22
4 (medium-high)	297 (14.2)	1990 (19.1)	2287 (18.3)		0.13	0.25
5 (high)	340 (16.3)	2083 (20.0)	2423 (19.4)		0.10	0.23
Missing	38 (1.8)	26 (0.2)	64 (0.5)		0.16	0.03
Rural residence						
Missing	6 (0.3)	26 (0.2)	32 (0.3)	<.001	0.01	0.17
No	1977 (94.8)	9395 (90.1)	11 372 (90.9)		0.18	0.36
Yes	102 (4.9)	1004 (9.6)	1106 (8.8)		0.18	0.37
Migrant status						
Canadian born	1851 (88.8)	8653 (83.0)	10 504 (84.0)	<.001	0.17	0.28
Immigrant	188 (9.0)	1481 (14.2)	1669 (13.3)		0.16	0.30
Refugee	46 (2.2)	291 (2.8)	337 (2.7)		0.04	0.25
**Clinical characteristics**						
Ontario drug benefit coverage^b^						
No	982 (47.1)	7998 (76.7)	8980 (71.8)	<.001	0.64	0.14
Yes	1103 (52.9)	2427 (23.3)	3530 (28.2)		0.64	0.14
Clinical comorbidities						
Rheumatoid arthritis	6 (0.3)	46 (0.4)	52 (0.4)	.32	0.03	0.31
Asthma	489 (23.5)	2034 (19.5)	2523 (20.2)	<.001	0.10	0.17
Cancer	28 (1.3)	116 (1.1)	144 (1.2)	.37	0.02	0.17
Chronic heart failure	8 (0.4)	22 (0.2)	30 (0.2)	.14	0.03	0.11
Crohn disease or colitis	10 (0.5)	61 (0.6)	71 (0.6)	.56	0.01	0.24
COPD	51 (2.4)	156 (1.5)	207 (1.7)	.002	0.07	0.12
Diabetes	115 (5.5)	352 (3.4)	467 (3.7)	<.001	0.10	0.13
HIV	34 (1.6)	12 (0.1)	46 (0.4)	<.001	0.16	0.01
Hypertension	128 (6.1)	644 (6.2)	772 (6.2)	.95	0	0.20
Myocardial infarction	6 (0.3)	28 (0.3)	34 (0.3)	.88	0	0.19
Total comorbidity count						
Mean (SD)	0.42 (0.67)	0.33 (0.60)	0.35 (0.61)	<.001	0.14	1.26
Median (IQR)	0 (0-1)	0 (0-1)	0 (0-1)	<.001	0.13	1.15
0	1383 (66.3)	7484 (71.8)	8867 (70.9)	<.001	0.12	0.18
1	565 (27.1)	2544 (24.4)	3109 (24.9)		.06	0.19
≥2	137 (6.6)	397 (3.8)	534 (4.3)		.12	0.12
Psychosocial ADGs						
0, Absence	494 (23.7)	8041 (77.1)	8535 (68.2)	<.001	1.26	0.20
ADG23, psychosocial: time limited, minor	380 (18.2)	381 (3.7)	761 (6.1)	<.001	0.48	0.05
ADG24, psychosocial: recurrent or persistent, stable	1514 (72.6)	2127 (20.4)	3641 (29.1)	<.001	1.23	0.16
ADG25, psychosocial: recurrent or persistent, unstable	487 (23.4)	388 (3.7)	875 (7.0)	<.001	0.60	0.04
Nonpsychosocial ADGs						
0, Absence	1089 (52.2)	7457 (71.5)	8546 (68.3)	<.001	0.41	0.16
ADG3, time limited: major	122 (5.9)	315 (3.0)	437 (3.5)	<.001	0.14	0.11
ADG4, time limited: major primary infections	221 (10.6)	694 (6.7)	915 (7.3)	<.001	0.14	0.13
ADG9, likely to recur: progressive	20 (1.0)	71 (0.7)	91 (0.7)	.17	0.03	0.14
ADG11, chronic medical: unstable	528 (25.3)	839 (8.0)	1367 (10.9)	<.001	0.48	0.08
ADG16, chronic specialty: unstable orthopedic	21 (1.0)	85 (0.8)	106 (0.8)	.38	0.02	0.16
ADG22, injuries or adverse effects: major	481 (23.1)	1504 (14.4)	1985 (15.9)	<.001	0.22	0.14
ADG32, malignant neoplasms	67 (3.2)	287 (2.8)	354 (2.8)	.25	0.03	0.17

Abbreviations: ADG, Aggregate Diagnosis Group; COPD, chronic obstructive pulmonary disease; IQR, interquartile range; SMD, standardized mean difference.

^a^ Income quintiles (source: Census data from Statistics Canada) are derived from the household after-tax income per single person equivalent (ie, a measure that takes into account the economies of scale, so that a 2-person household counts as 1.24 single-person equivalents). Income quintiles are also calculated within each census unit to account for differences in cost of living, so that 2 individuals with the same income who live in different metropolitan areas may be grouped in different quintiles.

^b^ Based on whether an outpatient drug prescription was filled.

**Table 2.** Age Distribution of Transgender Individuals by Gender Identity Category

	No. (%)				
	Nonbinary (n = 130)	Transgender		Unknown (n = 346)	Total (N = 2008)
		Man (n = 761)	Woman (n = 771)		
Age, y					
Mean (SD)	28.75 (11.29)	28.95 (11.94)	31.36 (13.08)	32.06 (14.17)	30.40 (12.81)
Median (IQR)	26 (21-34)	25 (20-34)	27 (21-38)	30 (22-41)	26 (21-37)
≤24	59 (45.4)	357 (47.2)	308 (40.3)	170 (39.3)	894 (42.9)
25-44	57 (43.8)	311 (41.1)	330 (43.1)	184 (42.5)	882 (42.3)
45-64	13 (10.0)	81 (10.7)	111 (14.5)	71 (16.4)	276 (13.2)
≥65^a^	NA	NA	NA	NA	33 (1.6)

Abbreviation: IQR, interquartile range; NA, not applicable.

^a^ Denotes suppressed values because of small number of participants in this category.

Transgender individuals were more likely to live in the 2 lowest neighborhood income quintiles compared with matched controls (lowest-income quintile: 625 [30.0%] vs 2197 [21.1%]; *P* < .001). Most transgender individuals resided in urban settings (1977 individuals [94.8%]), similar to the general population (11 372 [90.1%]). There was a higher proportion of transgender individuals covered under the public provincial drug plan compared with the matched controls (1103 [52.9%] vs 2427 [23.3%]; *P* < .001).

Transgender individuals had higher rates of asthma (489 [23.5%] vs 2034 [19.5%]; *P* < .001), diabetes (115 [5.5%] vs 352 [3.4%]; *P* < .001), COPD (51 [2.4%] vs 156 [1.5%]; *P* = .002), and HIV (34 [1.6%] vs 12 [0.1%]; *P* < .001) compared with cisgender controls. Clinical chronic comorbidities were higher among the transgender population compared with the general population (702 [33.7%] vs 2941 [28.2%]; *P* < .001). Transgender individuals also had a higher prevalence of comorbidity as measured by ADGs, including a higher prevalence of both medical ADGs and psychosocial ADGs, the latter reflecting a greater burden of psychiatric comorbidity. For example, psychiatric comorbidities were reported in 2384 (22.9%) in the general population (*P* < .001) compared with 1591 transgender individuals (76.3%).

Table 3 presents health service use among transgender and age-matched cisgender individuals. Overall health service use was significantly higher among transgender individuals (eTable 1 in the Supplement). Transgender individuals had a higher mean (SD) number of mental health–related primary care physician visits (9.11 [20.19] vs 2.10 [13.44]; SMD, 2.26) and non–mental health–related primary care physician visits (13.44 [14.66] vs 11.08 [13.19]; SMD, 1.24), psychiatrist visits (8.25 [23.13] vs 0.93 [9.57]; SMD, 5.84), and nonpsychiatric specialist visits (eg, endocrinologist visits: 0.92 [2.35] vs 0.13 [0.94]; SMD, 6.21) compared with the general population. Total mean (SD) ED visits were higher among transgender individuals (4.66 [11.05] vs 1.88 [3.95]; SMD, 7.84), particularly for mental health (1.22 [5.18] vs 0.11 [0.76]; SMD, 46.28) and self-harm (0.13 [0.91] vs 0.01 [0.36]; SMD, 6.53) compared with the matched controls. Transgender individuals also had higher mean (SD) rates of hospitalization compared with the general population (0.77 [2.25] vs 0.23 [0.77]; SMD, 8.41), especially for mental health–related reasons (0.53 [1.87] vs 0.03 [0.33]; SMD, 31.4).

**Table 3.** Comparative Health Service Use Between Transgender and Age-Matched Cisgender Individuals From January 2012 to December 2016

	Transgender individuals (n = 2085)	Cisgender individuals (n = 10 425)	Total (N = 12 510)	*P* value	SMD	Variance ratio
**Physician visits**						
Overall PCP visits						
Mean (SD)	22.55 (27.15)	13.18 (19.62)	14.74 (21.35)	.40	1.91	<.001
Median (IQR)	16 (8-28)	8 (3-17)	9 (4-19)	.60	0.92	<.001
Sum	47 026	137 414	184 440	NA	NA	NA
MH PCP visits						
Mean (SD)	9.11 (20.19)	2.10 (13.44)	3.27 (15.01)	.41	2.26	<.001
Median (IQR)	4 (1-10)	0 (0-1)	0 (0-2)	1.33	1.12	<.001
Sum	19 000	21 897	40 897	NA	NA	NA
Non-MH PCP visits						
Mean (SD)	13.44 (14.66)	11.08 (13.19)	11.47 (13.47)	.17	1.24	<.001
Median (IQR)	9 (4-18)	8 (3-15)	8 (3-16)	.22	0.93	<.001
Sum	28 026	115 517	143 543	NA	NA	NA
Total psychiatrist visits						
Mean (SD)	8.25 (23.13)	0.93 (9.57)	2.15 (13.15)	.41	5.84	<.001
Median (IQR)	1 (0-6)	0 (0-0)	0 (0-0)	1.23	3.26	<.001
Sum	17 210	9691	26 901	NA	NA	NA
Total urologist visits						
Mean (SD)	0.29 (1.47)	0.13 (0.95)	0.16 (1.06)	.13	2.37	<.001
Median (IQR)	0 (0-0)	0 (0-0)	0 (0-0)	.17	1.88	<.001
Sum	605	1354	1959	NA	NA	NA
Total plastic surgeon visits						
Mean (SD)	0.38 (1.16)	0.16 (0.90)	0.20 (0.95)	.21	1.68	<.001
Median (IQR)	0 (0-0)	0 (0-0)	0 (0-0)	.32	2.35	<.001
Sum	787	1698	2485	NA	NA	NA
Total OBGYN visits						
Mean (SD)	0.66 (2.49)	1.21 (5.23)	1.12 (4.89)	.13	0.23	<.001
Median (IQR)	0 (0-0)	0 (0-0)	0 (0-0)	.02	0.98	0.482
Sum	1366	12 583	13 949	NA	NA	NA
Total cardiologist visits						
Mean (SD)	0.18 (2.02)	0.12 (1.16)	0.13 (1.34)	.04	3.06	0.06
Median (IQR)	0 (0-0)	0 (0-0)	0 (0-0)	.08	1.39	<.001
Sum	366	1200	1566	NA	NA	NA
Total endocrinologist visits						
Mean (SD)	0.92 (2.35)	0.13 (0.94)	0.26 (1.32)	.44	6.21	<.001
Median (IQR)	0 (0-0)	0 (0-0)	0 (0-0)	.57	5.23	<.001
Sum	1911	1325	3236	NA	NA	NA
**Total ED visits**						
Overall visits						
Mean (SD)	4.66 (11.05)	1.88 (3.95)	2.35 (5.86)	.33	7.84	<.001
Median (IQR)	2 (0-5)	1 (0-2)	1 (0-3)	.50	1.14	<.001
Sum	9717	19 621	29 338	NA	NA	NA
MH ED visits						
Mean (SD)	1.22 (5.18)	0.11 (0.76)	0.30 (2.26)	.30	46.28	<.001
Median (IQR)	0 (0-1)	0 (0-0)	0 (0-0)	.69	4.2	<.001
Sum	2536	1174	3710	NA	NA	NA
SH ED visits						
Mean (SD)	0.13 (0.91)	0.01 (0.36)	0.03 (0.50)	.18	6.53	<.001
Median (IQR)	0 (0-0)	0 (0-0)	0 (0-0)	.34	8.82	<.001
Sum	279	135	414	NA	NA	NA
Non-MH ED visits						
Mean (SD)	3.31 (6.67)	1.76 (3.49)	2.02 (4.23)	.29	3.66	<.001
Median (IQR)	1 (0-4)	1 (0-2)	1 (0-2)	.36	1.12	<.001
Sum	6902	18 312	25 214	NA	NA	NA
**Total No. of hospitalizations**						
Overall hospitalizations						
Mean (SD)	0.77 (2.25)	0.23 (0.77)	0.32 (1.18)	.32	8.41	<.001
Median (IQR)	0 (0-1)	0 (0-0)	0 (0-0)	.39	1.84	<.001
Sum	1602	2366	3968	NA	NA	NA
MH hospitalizations						
Mean (SD)	0.53 (1.87)	0.03 (0.33)	0.12 (0.84)	.37	31.4	<.001
Median (IQR)	0 (0-0)	0 (0-0)	0 (0-0)	.60	8.64	<.001
Sum	1105	357	1462	NA	NA	NA
Non-MH hospitalizations						
Mean (SD)	0.24 (1.11)	0.19 (0.67)	0.20 (0.76)	.05	2.81	0.012
Median (IQR)	0 (0-0)	0 (0-0)	0 (0-0)	.03	1.06	0.216
Sum	497	2009	2506	NA	NA	NA

Abbreviations: ED, emergency department; IQR, interquartile range; MH, mental health; OBGYN, obstetrician-gynecologist; PCP, Primary Care Provider; SH, self-harm; SMD, standardized mean difference.

eTable 2 in the Supplement presents health service use among transgender individuals by gender identity category. Overall health service use was similar among all groups of transgender individuals. Total mean (SD) mental health–related primary care physician visits were numerically higher among transgender women compared with the overall transgender sample (10.11 [23.16] vs 9.11 [20.19]; *P* = .41), whereas non–mental health–related primary care physician visits were slightly numerically higher among transgender men compared with the overall transgender sample (14.57 [16.72] vs 13.44 [14.66]; *P* = .17), but neither difference was statistically significance. There were minimal differences for specialist visits, ED visits, and hospitalizations among the transgender sample.

## Discussion

We found that transgender individuals were more likely to live in lower-income neighborhoods, experience chronic physical and mental health conditions, and have higher health service use compared with the general population. Specifically, our study found higher rates of asthma, COPD, diabetes, and HIV and greater rates of mental health comorbidity. Transgender individuals were much more likely to see their primary care physician, visit the ED, and be admitted to the hospital, particularly for mental health and self-harm–related reasons, compared with the general population.

Transgender individuals frequently face discrimination, stigma, violence, and barriers to housing, employment, and education.^1,22^ A 2019 US study^23^ reported that transgender individuals have especially high rates of poverty (29.4%) compared with cisgender individuals (15.7%), which is further affected by racial and sexual orientation minority statuses. Our results show a similar trend, including potential barriers experienced by transgender individuals, such as a higher proportion of them receiving public drug coverage (a marker of low income, as public drug coverage is based on financial need) and living in lower-income neighborhoods compared with the general population. This disparity can also increasingly contribute to the adverse health circumstances and high rates of suicide experienced by transgender individuals. A 2015 survey-based study^24^ investigating the health and well-being of 433 transgender individuals in Ontario reported that 77% of transgender individuals had seriously considered suicide and 43% had attempted suicide.

Similar to our findings, previous studies have reported that transgender individuals experience a wide range of health disparities and poor health outcomes compared with their cisgender counterparts.^2,4,9,25,26^ For example, Proctor et al^27^ identified transgender Medicare beneficiaries in the US by using a variety of claims information, including hormone prescriptions and gender identity disorder diagnosis codes, and found that transgender individuals experienced high rates of depression, anxiety disorders, posttraumatic stress disorder, schizophrenia, and other psychotic disorders. Transgender individuals experience major challenges accessing health care because of a lack of transgender-competent and inclusive health services, lack of practitioners with sufficient transgender inclusion training, and systemic discrimination in primary health services and hospitals.^3,10,28^

An Ontario-based study^14^ found that 21% of transgender individuals reported avoiding the ED when they needed it because of previous stigma and discrimination based on their gender identity. While previous research has indicated that transgender individuals may avoid health services,^29,30,31^ our study shows transgender individuals use health services at much higher rates than the general population. We assessed transgender individuals who were attached to primary care, some of whom may have been actively medically transitioning either through transition-related surgery and/or hormone therapy, which may explain primary care physician visits. However, the high rates of ED visits are worrisome in the context of primary care physician attachment, especially given that all participating clinics specialized in transgender health. The higher rates of health service use for mental health and self-harm–related reasons may reflect the health service manifestation of stigma and discrimination.

The transgender sample identified in our study was relatively young, consistent with previous research, which has reported that transgender individuals are more likely to be younger compared with the overall population.^32,33^ Future efforts should be made to improve identification of older transgender individuals. Future studies should also investigate the health service use and health outcomes among transgender individuals living in small town and rural settings. Additionally, future research should examine how medical transition status (hormones and/or transition-related surgery) affects mental health outcomes and health service use, particularly mental health and self-harm–related ED visits and hospitalizations.

### Strengths and Limitations

A major strength of our study is the large sample of transgender individuals we were able to identify and link to administrative data. The size of our sample allowed us to assess the sociodemographic characteristics, chronic health conditions, and health service use of transgender individuals compared with the general population as well as compare these characteristics with data on health service use among gender identity categories, an area that has not been previously assessed.

Our study has several limitations. The participating health clinics were located in larger cities across Ontario, resulting in a sample of transgender individuals mainly located in urban settings, with minimal representation of transgender individuals living in small town and rural settings. We are unable to ascertain the degree to which our sample, based on the 4 clinics, is representative of the regional distribution of transgender individuals in Ontario. This may have limited the generalizability of the findings. Even though the transgender sample comprised individuals who self-identified as transgender, there is a chance that transgender individuals were included in the comparison sample, because we did not identify all of the transgender people in Ontario. Additionally, the Ontario Drug Benefit Database only included individuals who filled an outpatient drug prescription.

## Conclusions

This study found that transgender individuals tend to live in lower-income neighborhoods and experience higher rates of chronic conditions, including HIV, diabetes, asthma, COPD, and mental health comorbidities, compared with the general population. We also found that transgender individuals have significantly higher rates of health service use, particularly for mental health and self-harm–related reasons, even though they were connected to a primary care clinic specializing in transgender health.

Our study highlights the need to better understand the factors underlying the health disparities and high rates of mental health comorbidities experienced by transgender individuals. It demonstrates the importance of collecting gender identity information and the need for health care professionals to be attuned to the high potential for mental health issues and self-harm among transgender individuals. Efforts to improve the capacity to identify transgender individuals in administrative health care data would help to understand their health needs and inform public health and health service interventions to reduce the high rates of disease burden.

## Notes

Supplement. **eTable 1.** Comparative Health Service Use Between Transgender and Age-Matched Cisgender Individuals From 2012 to 2016 **eTable 2.** Health Service Use of Transgender Individuals by Gender Identity Category From 2012 to 2016 **eAppendix 1.** Self-Reported Gender Identities **eAppendix 2.** ACGs and ADGs Information
