# Supplementary material for: Assessment of Health Conditions and Health Service Use Among Transgender Patients in Canada
Source: JAMA Netw Open. 2020 Aug 28;3(8):e2015036. doi: 10.1001/jamanetworkopen.2020.15036 (PMC7455854; doi:10.1001/jamanetworkopen.2020.15036)
Supplement: Supplement. — eTable 1. Comparative Health Service Use Between Transgender and Age-Matched Cisgender Individuals From 2012 to 2016 eTable 2. Health Service Use of Transgender Individuals by Gender Identity Category From 2012 to 2016 eAppendix 1. Self-Reported Gender Identities eAppendix 2. ACGs and ADGs Information [file jamanetwopen-3-e2015036-s001.pdf]

Supplementary Online Content

Abramovich A, de Oliveira C, Kiran T, et al. Assessment of health conditions and health service use among transgender patients in Canada. *JAMA Netw Open*. 2020;3(8):e2015036.doi:10.1001/jamanetworkopen.2020.15036

eTable 1. Comparative Health Service Use Between Transgender and Age-Matched Cisgender Individuals From 2012 to 2016
eTable 2. Health Service Use of Transgender Individuals by Gender Identity Category From 2012 to 2016
eAppendix 1. Self-Reported Gender Identities
eAppendix 2. ACGs and ADGs Information

This supplementary material has been provided by the authors to give readers additional information about their work.

© 2020 Abramovich A et al. *JAMA Network Open.*

eTable 1. Comparative health service use between transgender and age-matched cisgender individuals from January 2012 to December 2016						
	Transgender Individuals (n=2,085)	Cisgender Individuals (n=10,425)	Total (n=12,510)	P-Value	Standardized Difference	Variance Ratio
Physician visits						
Primary Care Provider (PCP) visits						
Overall						
Mean ± SD	22.55 ± 27.15	13.18 ± 19.62	14.74 ± 21.35	0.4	1.91	<.001
Median (IQR[a])	16 (8-28)	8 (3-17)	9 (4-19)	0.6	0.92	<.001
Sum	47,026	137,414	184,440			
0 visits	93 (4.5%)	1,219 (11.7%)	1,312 (10.5%)	0.27	0.48	<.001
01 - 02 visits	106 (5.1%)	1,059 (10.2%)	1,165 (9.3%)	0.19	0.38	
3+ Visits	1,886 (90.5%)	8,147 (78.1%)	10,033 (80.2%)	0.34	0.4	
Mental Health (MH) PCP visits						
Mean ± SD	9.11 ± 20.19	2.10 ± 13.44	3.27 ± 15.01	0.41	2.26	<.001
Median (IQR)	4 (1-10)	0 (0-1)	0 (0-2)	1.33	1.12	<.001
Sum	19,000	21,897	40,897			
0 visits	401 (19.2%)	7,320 (70.2%)	7,721 (61.7%)	1.19	0.27	<.001
01 - 02 visits	387 (18.6%)	1,713 (16.4%)	2,100 (16.8%)	0.06	0.18	
3+ Visits	1,297 (62.2%)	1,392 (13.4%)	2,689 (21.5%)	1.17	0.1	
Non-MH PCP visits						
Mean ± SD	13.44 ± 14.66	11.08 ± 13.19	11.47 ± 13.47	0.17	1.24	<.001
Median (IQR)	9 (4-18)	8 (3-15)	8 (3-16)	0.22	0.93	<.001
Sum	28,026	115,517	143,543			
0 visits	135 (6.5%)	1,292 (12.4%)	1,427 (11.4%)	0.2	0.36	<.001
01 - 02 visits	215 (10.3%)	1,164 (11.2%)	1,379 (11.0%)	0.03	0.21	
3+ Visits	1,735 (83.2%)	7,969 (76.4%)	9,704 (77.6%)	0.17	0.26	
Total Psychiatrist visits						
Mean ± SD	8.25 ± 23.13	0.93 ± 9.57	2.15 ± 13.15	0.41	5.84	<.001
Median (IQR)	1 (0-6)	0 (0-0)	0 (0-0)	1.23	3.26	<.001
Sum	17,210	9,691	26,901			
0 visits	873 (41.9%)	9,536 (91.5%)	10,409 (83.2%)	1.24	0.06	<.001
01 - 02 visits	465 (22.3%)	404 (3.9%)	869 (6.9%)	0.57	0.04	
3+ Visits	747 (35.8%)	485 (4.7%)	1,232 (9.8%)	0.84	0.04	
Total Urologist visits						
Mean ± SD	0.29 ± 1.47	0.13 ± 0.95	0.16 ± 1.06	0.13	2.37	<.001
Median (IQR)	0 (0-0)	0 (0-0)	0 (0-0)	0.17	1.88	<.001
Sum	605	1,354	1,959			
0 visits	1,909 (91.6%)	9,973 (95.7%)	11,882 (95.0%)	0.17	0.11	<.001
01 - 02 visits	98 (4.7%)	278 (2.7%)	376 (3.0%)	0.11	0.12	
3+ Visits	78 (3.7%)	174 (1.7%)	252 (2.0%)	0.13	0.09	
Total Plastic Surgeon visits						
Mean ± SD	0.38 ± 1.16	0.16 ± 0.90	0.20 ± 0.95	0.21	1.68	<.001
Median (IQR)	0 (0-0)	0 (0-0)	0 (0-0)	0.32	2.35	<.001

© 2020 Abramovich A et al. *JAMA Network Open.*

Sum	787	1,698	2,485			
0 visits	1,746 (83.7%)	9,789 (93.9%)	11,535 (92.2%)	0.33	0.08	<.001
01 - 02 visits	239 (11.5%)	424 (4.1%)	663 (5.3%)	0.28	0.08	
3+ Visits	100 (4.8%)	212 (2.0%)	312 (2.5%)	0.15	0.09	
**Total OBGYN visits**						
Mean ± SD	0.66 ± 2.49	1.21 ± 5.23	1.12 ± 4.89	0.13	0.23	<.001
Median (IQR)	0 (0-0)	0 (0-0)	0 (0-0)	0.02	0.98	0.482
Sum	1,366	12,583	13,949			
0 visits	1,723 (82.6%)	8,758 (84.0%)	10,481 (83.8%)	0.04	0.19	<.001
01 - 02 visits	184 (8.8%)	629 (6.0%)	813 (6.5%)	0.11	0.14	
3+ Visits	178 (8.5%)	1,038 (10.0%)	1,216 (9.7%)	0.05	0.23	
**Total Cardiologist visits**						
Mean ± SD	0.18 ± 2.02	0.12 ± 1.16	0.13 ± 1.34	0.04	3.06	0.06
Median (IQR)	0 (0-0)	0 (0-0)	0 (0-0)	0.08	1.39	<.001
Sum	366	1,200	1,566			
0 visits	1,962 (94.1%)	9,992 (95.8%)	11,954 (95.6%)	0.08	0.14	<.001
01 - 02 visits	97 (4.7%)	307 (2.9%)	404 (3.2%)	0.09	0.13	
3+ Visits	26 (1.2%)	126 (1.2%)	152 (1.2%)	0	0.19	
**Total Endocrinologist visits**						
Mean ± SD	0.92 ± 2.35	0.13 ± 0.94	0.26 ± 1.32	0.44	6.21	<.001
Median (IQR)	0 (0-0)	0 (0-0)	0 (0-0)	0.57	5.23	<.001
Sum	1,911	1,325	3,236			
0 visits	1,641 (78.7%)	10,073 (96.6%)	11,714 (93.6%)	0.57	0.04	<.001
01 - 02 visits	171 (8.2%)	173 (1.7%)	344 (2.7%)	0.31	0.04	
3+ Visits	273 (13.1%)	179 (1.7%)	452 (3.6%)	0.45	0.03	
**Total Emergency Department (ED) visits**						
**Overall**						
Mean ± SD	4.66 ± 11.05	1.88 ± 3.95	2.35 ± 5.86	0.33	7.84	<.001
Median (IQR)	2 (0-5)	1 (0-2)	1 (0-3)	0.5	1.14	<.001
Sum	9,717	19,621	29,338			
0 visits	564 (27.1%)	4,630 (44.4%)	5,194 (41.5%)	0.37	0.25	<.001
01 - 02 visits	629 (30.2%)	3,433 (32.9%)	4,062 (32.5%)	0.06	0.21	
3+ Visits	892 (42.8%)	2,362 (22.7%)	3,254 (26.0%)	0.44	0.14	
**MH ED visits**						
Mean ± SD	1.22 ± 5.18	0.11 ± 0.76	0.30 ± 2.26	0.3	46.28	<.001
Median (IQR)	0 (0-1)	0 (0-0)	0 (0-0)	0.69	4.2	<.001
Sum	2,536	1,174	3,710			
0 visits	1,451 (69.6%)	9,835 (94.3%)	11,286 (90.2%)	0.68	0.05	<.001
01 - 02 visits	381 (18.3%)	483 (4.6%)	864 (6.9%)	0.44	0.06	
3+ Visits	253 (12.1%)	107 (1.0%)	360 (2.9%)	0.46	0.02	
**Self-Harm (SH) ED visits**						
Mean ± SD	0.13 ± 0.91	0.01 ± 0.36	0.03 ± 0.50	0.18	6.53	<.001
Median (IQR)	0 (0-0)	0 (0-0)	0 (0-0)	0.34	8.82	<.001
Sum	279	135	414			
0 visits	1,933 (92.7%)	10,344 (99.2%)	12,277 (98.1%)	0.34	0.02	<.001

© 2020 Abramovich A et al. *JAMA Network Open.*

1+ visits	152 (7.3%)	81 (0.7%)	233 (1.9%)	0.31	0.02	
**Non-MH ED visits**						
Mean ± SD	3.31 ± 6.67	1.76 ± 3.49	2.02 ± 4.23	0.29	3.66	<.001
Median (IQR)	1 (0-4)	1 (0-2)	1 (0-2)	0.36	1.12	<.001
Sum	6,902	18,312	25,214			
0 visits	665 (31.9%)	4,725 (45.3%)	5,390 (43.1%)	0.28	0.23	<.001
01 - 02 visits	674 (32.3%)	3,467 (33.3%)	4,141 (33.1%)	0.02	0.2	
3+ Visits	746 (35.8%)	2,233 (21.4%)	2,979 (23.8%)	0.32	0.15	
**Total number of hospitalizations**						
**Overall**						
Mean ± SD	0.77 ± 2.25	0.23 ± 0.77	0.32 ± 1.18	0.32	8.41	<.001
Median (IQR)	0 (0-1)	0 (0-0)	0 (0-0)	0.39	1.84	<.001
Sum	1,602	2,366	3,968			
0 visits	1,472 (70.6%)	8,946 (85.8%)	10,418 (83.3%)	0.37	0.12	<.001
01 - 02 visits	449 (21.5%)	1,318 (12.6%)	1,767 (14.1%)	0.24	0.13	
3+ Visits	164 (7.9%)	161 (1.5%)	325 (2.6%)	0.3	0.04	
**MH hospitalizations**						
Mean ± SD	0.53 ± 1.87	0.03 ± 0.33	0.12 ± 0.84	0.37	31.4	<.001
Median (IQR)	0 (0-0)	0 (0-0)	0 (0-0)	0.6	8.64	<.001
Sum	1,105	357	1,462			
0 visits	1,674 (80.3%)	10,227 (98.1%)	11,901 (95.1%)	0.6	0.02	<.001
01 - 02 visits	288 (13.8%)	160 (1.5%)	448 (3.6%)	0.47	0.03	
3+ Visits	123 (5.9%)	38 (0.4%)	161 (1.3%)	0.32	0.01	
**Non-MH hospitalizations**						
Mean ± SD	0.24 ± 1.11	0.19 ± 0.67	0.20 ± 0.76	0.05	2.81	0.012
Median (IQR)	0 (0-0)	0 (0-0)	0 (0-0)	0.03	1.06	0.216
Sum	497	2,009	2,506			
0 visits	1,799 (86.3%)	9,101 (87.3%)	10,900 (87.1%)	0.03	0.19	0.311
01 - 02 visits	256 (12.3%)	1,206 (11.6%)	1,462 (11.7%)	0.02	0.19	
3+ Visits	30 (1.4%)	118 (1.1%)	148 (1.2%)	0.03	0.16	

[a] Interquartile range (IQR).

© 2020 Abramovich A et al. *JAMA Network Open.*

eTable 2. Health service use of transgender individuals by gender identity category from January 2012 to December 2016						
	**Non-Binary (n=130)**	**Transgender Man (n=761)**	**Transgender Woman (n=771)**	**Unknown (n=433)**	**Total (n=2,085)**	**P-Value**
**Total Primary Care Provider (PCP) visits**						
Mean ± SD	20.95 ± 25.83	23.43 ± 25.52	22.72 ± 27.37	21.21 ± 29.80	22.55 ± 27.15	0.504
Median (IQR[a])	16 (8-25)	17 (9-29)	17 (9-29)	13 (6-27)	16 (8-28)	0.004
0 visits	7 (5.4%)	27 (3.6%)	29 (3.8%)	30 (6.9%)	93 (4.5%)	0.003
1+ visit	123 (94.6%)	730 (96.4%)	736 (96.2%)	403 (93.1%)	1,992 (95.5%)	
**Total Mental Health (MH) PCP visits**						
Mean ± SD	8.40 ± 19.98	8.86 ± 15.83	10.11 ± 23.16	8.00 ± 21.38	9.11 ± 20.19	0.325
Median (IQR)	4 (1-10)	5 (1-11)	5 (1-11)	3 (0-8)	4 (1-10)	<.001
0 visits	28 (21.5%)	126 (16.6%)	134 (17.5%)	113 (26.1%)	401 (19.2%)	<.001
1 visit	14 (10.8%)	77 (10.2%)	75 (9.8%)	58 (13.4%)	224 (10.7%)	
2+ Visits	88 (67.7%)	554 (73.2%)	556 (72.7%)	262 (60.5%)	1,460 (70.0%)	
**Total Non-MH PCP visits**						
Mean ± SD	12.55 ± 12.06	14.57 ± 16.72	12.61 ± 11.91	13.21 ± 15.81	13.44 ± 14.66	0.056
Median (IQR)	9 (5-17)	10 (5-19)	9 (4-17)	9 (4-18)	9 (4-18)	0.143
0 visits	9 (6.9%)	41 (5.4%)	43 (5.6%)	42 (9.7%)	135 (6.5%)	0.029
1+ visit	121 (93.1%)	716 (94.6%)	722 (94.4%)	391 (90.3%)	1,950 (93.5%)	
**Total Psychiatrist visits**						
Mean ± SD	8.53 ± 18.24	7.38 ± 19.54	8.13 ± 25.95	9.91 ± 24.86	8.25 ± 23.13	0.34
Median (IQR)	1 (0-7)	1 (0-5)	1 (0-5)	1 (0-6)	1 (0-6)	0.718
0 visits	57 (43.8%)	309 (40.8%)	300 (39.2%)	207 (47.8%)	873 (41.9%)	0.049
1 visit	15 (11.5%)	112 (14.8%)	121 (15.8%)	45 (10.4%)	293 (14.1%)	
2+ Visits	58 (44.6%)	336 (44.4%)	344 (45.0%)	181 (41.8%)	919 (44.1%)	
**Total Urologist visits**						
Mean ± SD	0.05 ± 0.39	0.31 ± 1.55	0.32 ± 1.60	0.28 ± 1.26	0.29 ± 1.47	0.257
Median (IQR)	0 (0-0)	0 (0-0)	0 (0-0)	0 (0-0)	0 (0-0)	0.027
0 visits	128 (98.5%)	695 (91.8%)	694 (90.7%)	392 (90.5%)	1,909 (91.6%)	0.084
1+ visit	*	*	*	*	176 (8.4%)	
**Total Plastic Surgeon visits**						
Mean ± SD	0.28 ± 0.86	0.52 ± 1.38	0.27 ± 0.93	0.35 ± 1.18	0.38 ± 1.16	<.001
Median (IQR)	0 (0-0)	0 (0-0)	0 (0-0)	0 (0-0)	0 (0-0)	0.002
0 visits	113 (86.9%)	605 (79.9%)	660 (86.3%)	368 (85.0%)	1,746 (83.7%)	0.004
1 visit	8 (6.2%)	67 (8.9%)	62 (8.1%)	29 (6.7%)	166 (8.0%)	
**Total OBGYN visits**						
Mean ± SD	0.72 ± 2.38	0.85 ± 3.24	0.52 ± 1.56	0.54 ± 2.32	0.66 ± 2.49	0.043
Median (IQR)	0 (0-0)	0 (0-0)	0 (0-0)	0 (0-0)	0 (0-0)	<.001

© 2020 Abramovich A et al. *JAMA Network Open.*

0 visits	109 (83.8%)	586 (77.4%)	642 (83.9%)	386 (89.1%)	1,723 (82.6%)	<.001
1+ visit	21 (16.2%)	171 (22.6%)	123 (16.1%)	47 (10.9%)	362 (17.4%)	
**Total Cardiologist visits**						
Mean ± SD	0.08 ± 0.31	0.24 ± 3.18	0.14 ± 0.85	0.16 ± 0.88	0.18 ± 2.02	0.75
Median (IQR)	0 (0-0)	0 (0-0)	0 (0-0)	0 (0-0)	0 (0-0)	0.818
0 visits	120 (92.3%)	713 (94.2%)	723 (94.5%)	406 (93.8%)	1,962 (94.1%)	0.115
1+ visit	10 (7.7%)	44 (5.8%)	42 (5.5%)	11 (6.2%)	123 (5.9%)	
**Total Endocrinologist visits**						
Mean ± SD	0.29 ± 1.10	1.03 ± 2.43	1.21 ± 2.60	0.39 ± 1.86	0.92 ± 2.35	<.001
Median (IQR)	0 (0-0)	0 (0-0)	0 (0-1)	0 (0-0)	0 (0-0)	<.001
0 visits	118 (90.8%)	569 (75.2%)	556 (72.7%)	398 (91.9%)	1,641 (78.7%)	<.001
1+ visit	12 (9.2%)	188 (24.8%)	209 (27.3%)	35 (8.1%)	444 (21.3%)	
**Total Emergency Department (ED) visits**						
Mean ± SD	3.67 ± 6.42	4.21 ± 9.44	4.95 ± 14.09	5.24 ± 8.28	4.66 ± 11.05	0.259
Median (IQR)	2 (0-4)	2 (0-4)	2 (0-5)	3 (0-7)	2 (0-5)	0.008
0 visits	43 (33.1%)	205 (27.1%)	204 (26.7%)	112 (25.9%)	564 (27.1%)	0.075
1 visit	22 (16.9%)	138 (18.2%)	139 (18.2%)	55 (12.7%)	354 (17.0%)	
2+ Visits	65 (50.0%)	414 (54.7%)	422 (55.2%)	266 (61.4%)	1,167 (56.0%)	
**Total MH ED visits**						
Mean ± SD	1.02 ± 2.93	1.06 ± 5.18	1.30 ± 6.07	1.41 ± 3.83	1.22 ± 5.18	0.639
Median (IQR)	0 (0-1)	0 (0-1)	0 (0-1)	0 (0-1)	0 (0-1)	0.459
0 visits	93 (71.5%)	534 (70.5%)	532 (69.5%)	292 (67.4%)	1,451 (69.6%)	0.733
1 visit	16 (12.3%)	97 (12.8%)	95 (12.4%)	51 (11.8%)	259 (12.4%)	
2+ Visits	21 (16.2%)	126 (16.6%)	138 (18.0%)	90 (20.8%)	375 (18.0%)	
**Total Self Harm (SH) ED visits**						
Mean ± SD	0.15 ± 0.55	0.10 ± 0.43	0.19 ± 1.39	0.09 ± 0.40	0.13 ± 0.91	0.123
Median (IQR)	0 (0-0)	0 (0-0)	0 (0-0)	0 (0-0)	0 (0-0)	0.345
0 visits	118 (90.8%)	706 (93.3%)	702 (91.8%)	407 (94.0%)	1,933 (92.7%)	0.528
1+ visit	12 (9.2%)	51 (6.7%)	63 (8.2%)	26 (6.0%)	152 (7.3%)	
**Total Non-MH ED visits**						
Mean ± SD	2.49 ± 3.87	3.05 ± 5.64	3.46 ± 8.30	3.74 ± 5.67	3.31 ± 6.67	0.154
Median (IQR)	1 (0-3)	1 (0-4)	1 (0-4)	2 (0-5)	1 (0-4)	0.029
0 visits	52 (40.0%)	240 (31.7%)	241 (31.5%)	132 (30.5%)	665 (31.9%)	0.021
1 visit	23 (17.7%)	160 (21.1%)	160 (20.9%)	64 (14.8%)	407 (19.5%)	
2+ Visits	55 (42.3%)	357 (47.2%)	364 (47.6%)	237 (54.7%)	1,013 (48.6%)	
**Total number of hospitalizations**						
Mean ± SD	0.69 ± 1.68	0.66 ± 1.94	0.78 ± 2.71	0.96 ± 1.97	0.77 ± 2.25	0.161
Median (IQR)	0 (0-1)	0 (0-1)	0 (0-1)	0 (0-1)	0 (0-1)	0.009

© 2020 Abramovich A et al. *JAMA Network Open.*

0 visits	94 (72.3%)	542 (71.6%)	555 (72.5%)	281 (64.9%)	1,472 (70.6%)	0.021
1 visit	18 (13.8%)	113 (14.9%)	115 (15.0%)	65 (15.0%)	311 (14.9%)	
2+ Visits	18 (13.8%)	102 (13.5%)	95 (12.4%)	87 (20.1%)	302 (14.5%)	
**Total MH hospitalizations**						
Mean ± SD	0.49 ± 1.53	0.45 ± 1.67	0.53 ± 2.13	0.68 ± 1.76	0.53 ± 1.87	0.26
Median (IQR)	0 (0-0)	0 (0-0)	0 (0-0)	0 (0-0)	0 (0-0)	0.055
0 visits	106 (81.5%)	614 (81.1%)	625 (81.7%)	329 (76.0%)	1,674 (80.3%)	0.058
1 visit	10 (7.7%)	78 (10.3%)	73 (9.5%)	43 (9.9%)	204 (9.8%)	
2+ Visits	14 (10.8%)	65 (8.6%)	67 (8.8%)	61 (14.1%)	207 (9.9%)	
**Total Non-MH hospitalizations**						
Mean ± SD	0.20 ± 0.56	0.21 ± 0.74	0.25 ± 1.54	0.29 ± 0.88	0.24 ± 1.11	0.659
Median (IQR)	0 (0-0)	0 (0-0)	0 (0-0)	0 (0-0)	0 (0-0)	0.388
0 visits	111 (85.4%)	656 (86.7%)	668 (87.3%)	364 (84.1%)	1,799 (86.3%)	0.471
1+ visit	19 (14.6%)	101 (13.3%)	97 (12.7%)	69 (15.9%)	286 (13.7%)	

* denotes suppressed values due to small cell counts.
[a] Interquartile range (IQR).

© 2020 Abramovich A et al. *JAMA Network Open.*

eAppendix 1

Self-Reported Gender Identities
Trans man,
Female to male,
Trans woman,
Male to female,
Non-binary,
Gender non-conforming,
Genderqueer,
Genderfluid,
2-spirit,
Androgynous,
Agender,
Queer

© 2020 Abramovich A et al. *JAMA Network Open.*

eAppendix 2

Adjusted Clinical Groups and Aggregated Diagnostic Groups Information

Johns Hopkins Adjusted Clinical Groups (ACGs) and Aggregated Diagnostic Groups (ADGs)

The Adjusted Clinical Group (ACG) scoring system was used to measure patients' comorbidity based on 2 years health service utilization prior to index.

ACGs are a person-focused diagnosis-based method of categorizing patients' illnesses. Over time, each person develops numerous conditions. Based on the pattern of these morbidities, the ACG approach assigns each individual to a single ACG category. Thus, an ACG captures the specific clustering of morbidities experienced by a person over a given period of time, such as a year.

The ACG System assigns all ICD (-9, -9-CM, -10) codes to one of 32 diagnosis clusters known as Aggregated Diagnosis Codes, or ADGs. Individual diseases or conditions are placed into a single ADG cluster based on five clinical dimensions:

• **Duration of the condition (acute, recurrent, or chronic):** How long will healthcare resources be required for the management of this condition?

• **Severity of the condition (e.g., minor and stable versus major and unstable):** How intensely must healthcare resources be applied to manage the condition?

• **Diagnostic certainty (symptoms versus documented disease):** Will a diagnostic evaluation be needed or will services for treatment be the primary focus?

• **Etiology of the condition (infectious, injury, or other):** What types of healthcare services will likely be used?

• **Specialty care involvement (e.g., medical, surgical, obstetric, hematology):** To what degree will specialty care services be required?

© 2020 Abramovich A et al. *JAMA Network Open.*
